# Supplementary material for: Saccharomyces boulardii (CNCM I-745) improves intestinal damage in sepsis by remodeling intestinal flora structure
Source: Front Cell Infect Microbiol. 2025 Jul 31;15:1602792. doi: 10.3389/fcimb.2025.1602792 (PMC12350329; doi:10.3389/fcimb.2025.1602792)
Supplement: Supplementary file 1 [file DataSheet1.zip › summary/summary/7_Differential_analysis/2_LEfSe_result/Group_DW4_vs_CW4_vs_BW4_vs_AW4_0.05_3.0/LEfSe_Group_DW4_vs_CW4_vs_BW4_vs_AW4.abund.pdf]

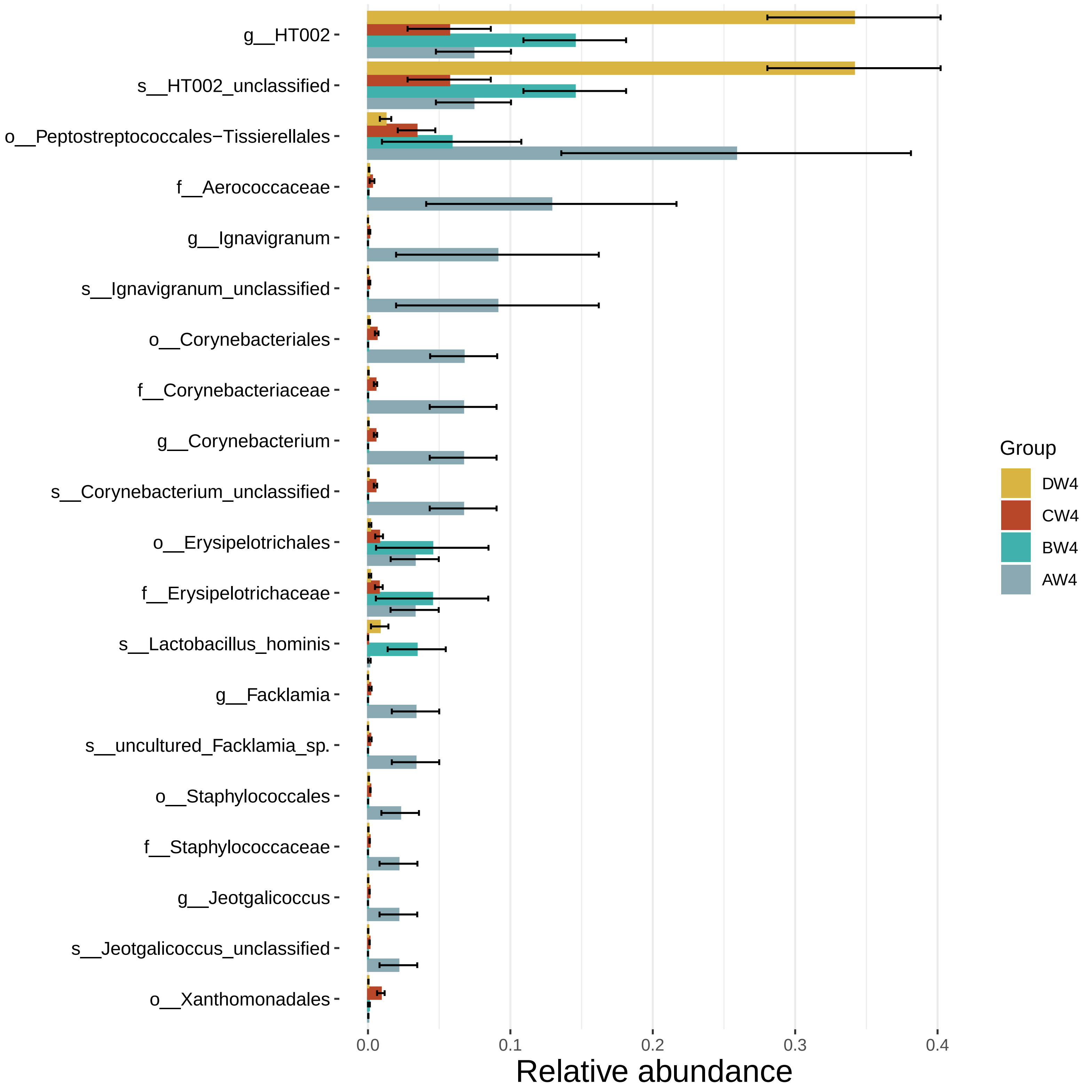
g__HT002
s__HT002_unclassified
o__Peptostreptococcales−Tissierellales
f__Aerococcaceae
g__Ignavigranum
s__Ignavigranum_unclassified
o__Corynebacteriales
f__Corynebacteriaceae
g__Corynebacterium
s__Corynebacterium_unclassified
o__Erysipelotrichales
f__Erysipelotrichaceae
s__Lactobacillus_hominis
g__Facklamia
s__uncultured_Facklamia_sp.
o__Staphylococcales
f__Staphylococcaceae
g__Jeotgalicoccus
s__Jeotgalicoccus_unclassified
o__Xanthomonadales
0.0
0.1
0.2
0.3
0.4
Relative abundance
Group
DW4
CW4
BW4
AW4
